# Supplementary material for: Application of Multi-Dimensional Intelligent Visual Quantitative Assessment System to Evaluate Hand Function Rehabilitation in Stroke Patients
Source: Brain Sci. 2022 Dec 10;12(12):1698. doi: 10.3390/brainsci12121698 (PMC9775443; doi:10.3390/brainsci12121698)
Supplement: Supplementary file 1 [file brainsci-12-01698-s001.zip › brainsci-2050099-supplementary.pdf]

**Supplementary Table S1.** Consistency of the MDIVQAS for repeated measures to assess hand function.

| Movement (n = 24) | First measurement | Second measurement | Third measurement | F | *p* |
|---|---|---|---|---|---|
| Wrist ulnar deviation | 23.00 (4.75 - 32.75) | 21.50 (6.25 - 32.75) | 19.50 (4.75 - 30.75) | 0.326 | 0.711 |
| Wrist dorsiextension | 35.50 (21.25 - 43.50) | 36.50 (15.75 - 43.50) | 32.50 (20.50 - 44.75) | 2.489 | 0.098 |
| Finger adduction and abduction | 113.00 (58.00 - 146.75) | 98.00 (62.25 - 149.25) | 119.00 (43.75 - 139.25) | 0.430 | 0.643 |
| Forearm pronation | 82.50 (25.50 - 88.00) | 83.00 (27.00 - 86.75) | 79.00 (22.00 - 87.00) | 0.976 | 0.374 |
| Forearm supination | 81.00 (13.00 - 88.00) | 79.00 (10.50 - 88.00) | 78.00 (14.50 - 88.00) | 1.666 | 0.204 |
| Cylindrical grip | 13.00 (4.50 - 19.75) | 12.50 (4.75 - 19.00) | 14.00 (5.25 - 21.00) | 2.858 | 0.071 |
| Spherical grip | 36.00 (7.00 - 69.50) | 39.00 (9.25 - 64.75) | 38.50 (5.00 - 65.75) | 0.214 | 0.753 |
| Thumb abduction | 15.00 (4.50 - 23.75) | 16.50 (3.75 - 22.75) | 17.00 (5.50 - 23.00) | 3.348 | 0.120 |
| Thumb flexion and extension | 44.00 (12.25 - 65.00) | 43.50 (9.25 - 62.75) | 41.00 (11.50 - 63.75) | 0.066 | 0.925 |
| Thumb rotation | 28.00 (10.00 - 39.75) | 24.00 (7.75 - 38.00) | 27.50 (10.00 - 37.25) | 3.603 | 0.045* |

Note:*$p < 0.05$.

**Supplementary Table S2.** Correlation between MDIVQAS and FMA-W/H, Brunnstrom (hand), and ARAT

| Evaluation methods | FMA-UE (n = 82) | FMA-W/H (n = 82) | Brunnstrom (hand) (n = 72) | ARAT (n = 36) |
|---|---|---|---|---|
| Multi-dimensional intelligent visual quantitative assessment system Correlation coefficient (R) | 0.690** | 0.796** | 0.895** | 0.747** |
| *p* | 0.000 | 0.000 | 0.000 | 0.000 |

Note:*$p < 0.05$,**$p < 0.01$.

**Supplementary Table S3.** Correlations between MDIVQAS and protractor measurement.

| Movement | r | *p* |
|---|---|---|
| Forearm pronation (n=81) | 0.974** | 0.000 |
| Forearm supination (n=81) | 0.973** | 0.000 |
| Ulnar deviation of wrist (n=81) | 0.763** | 0.000 |
| wrist dorsiextension (n=81) | 0.790** | 0.000 |
| Finger adduction and abduction1 (n=37) | 0.832** | 0.000 |
| Finger adduction and abduction2 (n=37) | 0.916** | 0.000 |
| Finger adduction and abduction3 (n=37) | 0.748** | 0.000 |
| Finger adduction and abduction4 (n=37) | 0.815** | 0.000 |
| Sum of finger adduction and abduction (n=37) | 0.908** | 0.000 |

Note: *$p < 0.05$,**$p < 0.01$; finger adduction abduction 1: angle between the thumb and index finger of the affected hand, finger adduction and abduction 2: angle between the index finger and the middle finger of the affected hand, finger adduction and abduction 3: angle between the middle finger and the ring finger of the affected hand, finger adduction and abduction 4: angle between the ring finger and the little finger of the affected hand.

**Supplementary Table S4.** Comparison of differences between FMA-UE, FMA-W/H, Brunnstrom and ARAT before and after treatment.

| | **Before the treatment (n = 46)** | **After treatment (n = 46)** | **t** | ***p* values** | **95%CI** |
|---|---|---|---|---|---|
| FMA-UE | 39.67±15.977 | 48.44±17.118 | -4.233** | 0.003 | -13.559～-3.996 |
| FMA-W/H | 14.00±6.500 | 17.78±7.965 | -3.640** | 0.007 | -6.171～-1.384 |
| Brunnstrom (hand) | 3.07±1.597 | 3.87±1.771 | -7.284** | 0.000 | -1.027～-7.284 |
| Brunnstrom (upper limb) | 3.46±1.361 | 4.24±1.369 | -7.622** | 0.000 | -0.989～-0.576 |
| ARAT | 40.00±17.692 | 44.00±18.486 | -2.502* | 0.037 | -7.686～-0.314 |

Note:*p < 0.05,**p < 0.01.

**Supplementary Table S5.** Comparison the differences of the AROM in finger adduction and abduction. With protractor before and after rehabilitation treatment

| **Movement** | **Before treatment (n = 37)** | **After treatment (n = 37)** | **Z** | ***p* values** |
|---|---|---|---|---|
| Finger adduction and abduction 1 | 10 (0 to 40) | 25 (0 to 48) | 3.157** | 0.002 |
| Finger adduction and abduction 2 | 5 (0 to 20) | 15 (0 to 23) | 2.905** | 0.004 |
| Finger adduction and abduction 3 | 5 (0 to 12.5) | 6 (0 to 17) | 2.766** | 0.006 |
| Finger adduction and abduction 4 | 2 (0 to 20) | 15 (0 to 22.5) | 2.747** | 0.006 |
| Sum of finger adduction and abduction | 32 (0 to 152) | 100 (0 to 166) | 3.971** | 0.000 |

Note:**p < 0.01, finger adduction abduction 1: angle between the thumb and index finger of the affected hand, finger adduction and abduction 2: angle between the index finger and the middle finger of the affected hand, finger adduction and abduction 3: angle between the middle finger and the ring finger of the affected hand, finger adduction and abduction 4: angle between the ring finger and the little finger of the affected hand. Z represents the effect size of the two-sample K-S test.

**Supplementary Table S6.** Comparison of differences before and after rehabilitation treatment using MDIVQAS

| **action** | **Before the treatment (n = 47)** | **After treatment (n = 47)** | **Z** | ***p* values** |
|---|---|---|---|---|
| Wrist ulnar deviation | 48.39 (14.78-65.58) | 46.15 (10.34-70.27) | 2.621** | 0.008 |
| Wrist dorsiextension | 47.21 (14.46 to 74.52) | 61.29 (20.41-84.62) | 2.961** | 0.002 |
| Forearm pronation | 91.48 (41.19 to 100.00) | 86.36 (40.91 ~ 100.00) | 2.233* | 0.024 |
| Forearm supination | 80.11 (13.75 to 100.00) | 88.75 (8.11 ~ 100.00) | 3.393** | 0.001 |
| Cylindrical grip | 46.59 (16.19 to 74.32) | 58.33 (23.53-83.87) | 3.852** | 0.000 |
| Spherical grip | 45.36 (11.42 to 75.55) | 69.40 (14.73-87.73) | 2.839** | 0.004 |
| Thumb abduction | 40.67 (20.63 to 68.34) | 58.97 (8.33-82.14) | 2.177* | 0.029 |
| Thumb flexion and extension | 95.09 (32.42 to 146.20) | 100.52 (12.55 ~ 159.70) | 2.788** | 0.005 |
| Thumb rotation | 54.25 (15.94 to 84.22) | 66.33 (15.93-85.54) | 2.904** | 0.004 |
| Finger adduction and abduction 1 | 31.82 (6.85 to 69.99) | 45.64 (12.44 ~ 91.48) | 3.077** | 0.002 |
| Finger adduction and abduction 2 | 25.00 (9.55 to 62.50) | 43.99 (13.84 ~ 83.75) | 2.178* | 0.029 |
| Finger adduction and abduction 3 | 25.93 (7.28 to 51.19) | 50.84 (8.08 to 69.11) | 3.115** | 0.002 |
| Finger adduction and abduction 4 | 32.50 (6.07 to 56.02) | 47.81 (12.26 ~ 68.61) | 2.510* | 0.012 |
| Sum of finger adduction and abduction | 195.47 (37.55 to 329.37) | 286.10 (53.59 ~ 400.46) | 3.554** | 0.000 |

Note:*p < 0.05,*p < 0.01.
